# Post-Licensure Geropsychology Consultation Program: Strengthening Pathways to Board Certification

**DOI:** 10.1093/geroni/igaf122.782

**Published:** 2025-12-31

**Authors:** Erin Kube, Michelle Mlinac, Lisa Lind, Dolores Gallagher Thompson, Tonita Wroolie

**Affiliations:** VISN 19 Clinical Resource Hub, Salt Lake City, Utah, United States; VA Boston Healthcare System, Boston, Massachusetts, United States; Deer Oaks Behavioral Health, San Antonio, Texas, United States; Dept. of Psychiatry & Behavioral Sciences, Stanford Univ. School of Medicine, Los Altos, California, United States; Department of Psychiatry and Behavioral Sciences, Stanford University School of Medicine, Stanford, California, United States

## Abstract

There are approximately 4500 licensed psychologists in the US. Of these, 4% are board certified and only 2% of those are boarded in geropsychology. This is in contrast with the growing numbers of older adults being seen in mental health settings. The American Board of Geropsychology (ABGERO) conducted a needs assessment of psychologists, sampled from various aging-related listservs/practice settings to clarify interest in and barriers to board certification in geropsychology. Results among 20 respondents indicated gaps in education, supervised training, and general understanding of the board certification process. For practicing psychologists who developed an interest in geropsychology only after becoming licensed, a substantial barrier to board certification is lack of supervised clinical training experiences with older adults at the graduate, internship, or postdoctoral level. This presentation will describe this committee’s efforts to remove these barriers and demystify the board-certification process. We will identify opportunities to increase the number of board certified specialists in geropsychology, specifically through creation of a year-long consultation program aimed at post-licensure psychologists to build competence in working with older adults. This process may serve as a model to other gerontological professions about how to build pathways for generalists to specialize in clinical aging care.

